# Limbal versus Pars plana extraction of posterior segment IOFBs using 23-gauge vitrectomy: anatomical and visual outcomes, and predictive factors for postoperative retinal detachment and poor visual prognosis

**DOI:** 10.1186/s40942-025-00701-5

**Published:** 2025-07-14

**Authors:** Abbas A. A. Hashem, Ahmad S. Khalil, Sherif A. Dabour, Wael M. El-Haig

**Affiliations:** Department of Ophthalmology, Faculty of Medicine, Zagazig University, Zagazig, Egypt

**Keywords:** Intraocular foreign bodies, Pars plana vitrectomy, Visual and anatomical outcomes, Predictive factors

## Abstract

**Background:**

Retained posterior segment intraocular foreign bodies (IOFBs) present a surgical challenge with potential for serious complications, including retinal detachment (RD) and vision loss. While pars plana vitrectomy (PPV) is the standard technique, there is no consensus regarding the optimal route for IOFB extraction, whether through a limbal incision or via the pars plana. This study aims to compare anatomical and visual outcomes between the two surgical approaches and to identify risk factors for postoperative RD and poor visual outcome.

**Methods:**

This prospective comparative study included 51 eyes with retained posterior segment IOFBs and an attached retina at presentation. Patients were randomly assigned to IOFB extraction via either a limbal incision (*n* = 26) or a pars plana incision (*n* = 25), following 23-gauge PPV. All patients were followed for a median of 12 months. Primary outcomes included the incidence of postoperative RD recorded over the entire first postoperative year and best-corrected visual acuity (BCVA) at one year. Univariate and multivariate logistic regression analyses were performed to identify predictors of RD and poor visual outcome, defined as BCVA worse than 0.8 logMAR.

**Results:**

Postoperative RD developed in 8 eyes (15.7%): 2 eyes (7.7%) in the limbal group and 6 eyes (24%) in the pars plana group (*P* = 0.14). Impacted IOFB (*P* = 0.042) and longer foreign body diameter (*P* = 0.032) were independent predictors of RD. Both groups showed significant improvement in BCVA from baseline (*P* < 0.001), with no significant difference between groups at any follow-up point. Poor visual outcome was independently associated with longer wound length (*P* = 0.011) and preoperative VA ≤ 1.6 logMAR (*P* = 0.005). The route of extraction was not a significant predictor of anatomical or functional outcome.

**Conclusions:**

IOFB extraction via the limbus or pars plana using 23-gauge PPV provides comparable anatomical and visual outcomes. Impacted IOFBs and larger foreign bodies increase the risk of postoperative RD, while wound length and poor baseline visual acuity are strong predictors of poor final visual outcome. Early recognition of these predictors is important for optimizing surgical planning and patient counseling.

## Introduction

Ocular injuries with retained posterior segment intraocular foreign body (IOFB) pose a complex surgical challenge due to the difficulties in extraction and associated ocular damage. The associated injuries may include cataract, vitreous hemorrhage, retinal detachment, endophthalmitis and can lead to visual loss [1, 2].

The management of these cases requires a meticulous approach to ensure successful removal of the IOFB while minimizing further ocular trauma. Several surgical approaches and modifications are utilized to extract IOFBs, primarily through either a scleral or limbal incision [3, 4]. Among these, 23-gauge pars plana vitrectomy (PPV) with IOFB removal via the pars plana is a commonly practiced approach [3]. Many surgical techniques for IOFB extraction via the corneal route have been also described in the literature. The iris shelf technique involves using the peripheral iris as temporary support to prevent the IOFB from slippage into the posterior segment during removal [5]. The forceps-assisted extraction (handshake technique) ensures careful manipulation of the IOFB while minimizing endothelial trauma [6, 7]. The intraocular lens (IOL) blocking technique utilizes the posterior chamber intraocular lens as a barrier to prevent IOFB displacement during extraction [8]. Additionally, in the magnetic conduction technique, an external magnet is used to magnetize the vitrectomy cutter, which lifts the IOFB from the retinal surface and guides it through the pupil into the anterior chamber, where it is then extracted with forceps through a corneal incision [9].

There is no universal consensus on whether the limbal or pars plana approach is superior for the extraction of retained posterior segment intraocular foreign bodies, as both approaches have advantages and limitations. This study aimed to assess and compare the anatomical and visual outcomes following the extraction of a retained posterior segment IOFBs through either a limbal incision or the pars plana, using 23-gauge microincision vitrectomy. It also aimed to identify predictors of postoperative retinal detachment and poor visual prognosis.

## Patients and methods

This prospective comparative study was conducted on patients admitted to the Department of Ophthalmology, Zagazig University, with a primary diagnosis of penetrating ocular injury and a retained posterior segment IOFB. Patients were enrolled in the study if they met the inclusion criteria and did not meet the exclusion criteria after primary wound repair. The study adhered to the tenets of the Declaration of Helsinki and received approval from the Institutional Review Board of the Faculty of Medicine, Zagazig University (Zagazig, Egypt) (IRB#: 9040-24-10-2021).

The study included eyes with a retained posterior segment IOFB associated with crystalline lens injury. Eyes were excluded if they had a retained posterior segment IOFB associated with traumatic retinal detachment, a clear lens or were pseudophakic. Additionally, patients with severely damaged eyes presenting with no light perception, severely traumatized corneas impairing visualization during vitrectomy or endophthalmitis at initial presentation were excluded.

Patients were randomly assigned to either a limbal group, undergoing IOFB extraction through a limbal incision, or a pars plana group, undergoing extraction through a scleral incision. Patients were randomly allocated to one of the two surgical groups using the CDC Epi Info™ randomization tool, which generated a simple random sequence of numbers. Randomization was performed only after confirming that patients met all inclusion criteria and did not meet any of the exclusion criteria. All patients underwent a comprehensive ocular examination, including visual acuity assessment using Landolt C chart, and the values were converted to logMAR equivalents for statistical analysis. Anterior and posterior segment examinations were performed. Ocular ultrasonography was gently and carefully performed after primary globe repair over closed eyelids. Computed tomography of the orbit with 1-mm cuts was carried out to detect and localize the foreign body. Biometry for intraocular lens (IOL) power calculation was done preoperatively, before pars plana vitrectomy (PPV) and IOFB extraction. IOL power calculation was initially attempted using the IOL Master (Carl Zeiss Meditec); if optical biometry was not feasible due to media opacity, contact A-scan ultrasonography was used.

Planned surgeries were performed under general anesthesia 10–14 days after primary wound repair by SD and ASK, while in cases where endophthalmitis or secondary glaucoma developed during this deferment period, PPV and IOFB removal were scheduled for earlier surgical intervention.

A 2.8 mm limbal tunnel was created for lens management. If the anterior capsule tear was small, a continuous curvilinear capsulorhexis (CCC) was performed, followed by phacoaspiration of the lens material. In cases with an extensive anterior capsule tear, the capsule was trimmed before phacoaspiration. If the posterior capsule remained intact, a posterior capsulorhexis was performed in cases assigned to IOFB extraction via the limbus. However, in cases with a ruptured posterior capsule, a limited anterior vitrectomy was carried out, followed by posterior capsule trimming to eliminate vitreous traction and residual lens material, ensuring a stable intraocular environment for further surgical maneuvers.

A 23-gauge three-port PPV was performed using the R-Evolution CR platform (OPTIKON 2000, Inc., Rome, Italy) and the OPMI LUMERA 700 microscope (Carl Zeiss Meditec AG, Jena, Germany) with a wide-viewing system (Resight 700, Carl Zeiss Meditec AG, Jena, Germany). Trocar insertion was done transconjunctivally except in cases assigned to IOFB extraction via a pars plana incision, where a fornix-based conjunctival peritomy was created in the upper temporal quadrant (right eye) or upper nasal quadrant (left eye) to expose the sclera, followed by light cauterization before trocar insertion.

Core vitrectomy was performed, followed by posterior vitreous detachment (PVD) induction if it did not occur spontaneously. The IOFB was freed from any surrounding adhesions and if impacted it was surrounded by 2–3 rows of endolaser. The extraction approach varied based on the surgical plan. In the pars plana group, the trocar was removed, and the sclerotomy was enlarged according to IOFB size. The IOFB was grasped with intraocular forceps and extracted through the sclerotomy while an external earth magnet was applied near the incision to prevent slippage. In the limbal group, the anterior chamber was filled with viscoelastic, and the IOFB was transferred to the anterior iris surface using forceps. A second intraocular forceps introduced via the limbal incision was used for final extraction, with an external earth magnet applied near the limbus to prevent slippage.

. The final surgical steps included primary IOL implantation if capsular support was adequate. If capsular support was inadequate the eye was left aphakic. Scleral fixation of the intraocular lens (IOL) was then performed as a subsequent surgical procedure, either after ocular stabilization or at the time of silicone oil removal. Scleral fixation of an intraocular lens (IOL) was performed using either the Yamane technique or the four-flanged technique, depending on the availability of the IOL and IOL power. Eyes with intraoperative retinal breaks or subretinal fluid underwent fluid-air exchange followed by silicon oil (5000 cs) injection. Wound closure depended on the extraction approach. In the pars plana group, the extraction sclerotomy was closed with 7 − 0 vicryl, followed by conjunctival closure with 7 − 0 vicryl. In the limbal group, stromal hydration or 10 − 0 nylon suturing was performed as needed. Postoperatively all patients received topical antibiotics, steroids and cycloplegics for four weeks with gradual tapering. Systemic therapy consisted of oral ciprofloxacin 500 mg twice daily and non-steroidal anti-inflammatory agents for all cases, with systemic steroids administered when necessary.

### Statistical analysis

Data was coded and entered using the statistical package for the Social Sciences (SPSS) version 28 (IBM Corp., Armonk, NY, USA). Data was summarized using median and interquartile range in quantitative data and using frequency (count) and relative frequency (percentage) for categorical data. Data was double checked for normality using normality plots and Shapiro Wilk test and proved to be deviated from normal distribution. Comparisons between groups were done using the non-parametric Mann-Whitney U test [10]. For comparing categorical data, Chi square (χ2) test was performed. The exact test was used instead when the expected frequency is less than 5 [11]. Univariate and multivariate logistic regression were done to detect independent predictors of RD and poor VA [12]. P-values less than 0.05 were considered statistically significant.

## Results

Between January 2022 to September 2023 a total of 51 patients were enrolled in the study and were randomly allocated into two groups according to the planned site of extraction. The limbal group included 26 eyes and the pars plana group included 25 eyes. Forty-nine patients were males and two patients were females. The median age of patients was 26 years (IQR 19–39). Table 1 summarizes the baseline and perioperative characteristics of the enrolled patients. There was no statically significant difference between the two groups. Patients were followed for a median period of 12 months (IQR 12–18).

**Table 1:** Baseline and perioperative characteristics of the enrolled patients

Characteristic		Limbal group *N* = 26	Pars plana group *N* = 25	*P* value
**Age: median (IQR)**		**30.5 (23–36)**	**24 (18–39)**	**0.462**
**Mechanism of injury**	**Hammering**	**23 (88.5%)**	**24 (96.0%)**	**0.818**
	**Gunshot**	**2 (7.7%)**	**1 (4.0%)**	
	**Car accident**	**1 (3.8%)**	**0 (0.0%)**	
**Median preoperative VA (IQR)/ logMAR**		**1.4 (0.95–1.8)**	**1.3 (1-1.9)**	**0.820**
**Entry wound site**	**Cornea**	**23 (88.5%)**	**16 (64%)**	**0.101**
	**Corneo-sclera**	**1 (3.8%)**	**5 (20%)**	
	**Sclera**	**2 (7.7%)**	**4 (16%)**	
**Median wound length (IQR) / mm**		**3 (3 to 5)**	**3 (2 to 4)**	**0.153**
**Interval between primary repair to PPV and IOFB removal**	**Within 10–14 days**	**20 (76.9%)**	**18(72%)**	**0.687**
	**Less than 10–14 days**	**6 (23.1%)**	**7(28%)**	

Table 2 presents some intraoperative details recorded during 23-G PPV and IOFB extraction. In the pars plana group, the median time of the surgical procedure was significantly longer (˂0.001) while all other variables were statistically insignificant.

**Table 2:** Summary of some intra operative details during PPV and IOFB extraction

		Limbal group *N* = 26	Pars plana group *N* = 25	*P* value
**FB location**	**Vitreous**	**4 (15.38%)**	**5 (20%)**	**0.909**
	**Retinal surface**	**9 (34.61%)**	**8 (32%)**	
	**Impacted**	**13 (50%)**	**12 (48%)**	
**FB nature**	**Metallic**	**22 (84.6%)**	**23 (92%)**	**1**
	**Glass**	**1 (3.9%)**	**0 (0%)**	
	**Concrete**	**3 (11.5%)**	**2 (8%)**	
**Spontaneous PVD**		**16 (61.5%)**	**14 (56%)**	**0.688**
**Intra operative Complications**	**Retinal break**	**1 (3.8%)**	**3 (12%)**	**0.350**
	**Hemorrhage**	**2 (7.7%)**	**3 (12%)**	**0.668**
	**Localized RD**	**2 (7.7%)**	**3 (12%)**	**0.668**
**Tamponade**	**Saline**	**11 (42.3%)**	**11 (40%)**	**0.903**
	**Silicone oil**	**15 (57.69%)**	**14 (60%)**	
**IOL implantation**	**Yes**	**12 (46.15%)**	**16 (64%)**	**0.200**
	**No**	**14 (53.85%)**	**9 (36%)**	
**Median Time of surgery (IQR)/ min**		**45 (40–45)**	**50 (45–55)**	**< 0.001**
**Median FB longest diameter (IQR)/ mm**		**3 (2.88-5)**	**3 (2.5-4)**	**0.398**

### Anatomical outcomes

retinal detachment developed during the follow up period in 8 out the 51 eyes (15.68%), 2 eyes (7.7%) in the limbal group and 6 eyes (24%) in the pars plana group, yet the difference was not statistically significant (χ2 = 2.563, P value 0.140). The median time to develop postoperative retinal detachment was 2 months (IQR 1–2.25 months).

In a univariate logistic regression model to screen for predictors for the development of retinal detachment in the study eyes, seven potential independent variables were chosen: route of IOFB extraction (pars plana versus limbal route), presence of impacted FB, median foreign body longest diameter, FB entry wound site where corneal entry site was chosen as the reference category, absence of intraoperative posterior vitreous detachment (PVD) at the commencement of PPV, incidence of intraoperative complications and agent of endo-tamponade. The presence of impacted IOFB, *P* = 0.041, occurrence of intraoperative complications (iatrogenic retinal breaks and/or development of localized retinal detachment or intraoperative hemorrhage), *P* = 0.004 and median FB longest diameter, *P* = 0.021) were associated with a higher risk of developing RD. After subsequent analysis of the candidate predictors in stepwise multivariate logistic regression model the presence of impacted IOFB, *P* = 0.042 and FB longest diameter, *P* = 0.032 were significant risk factors for the development of postoperative retinal detachment (Table 3).

**Table 3:** Univariate and multivariate logistic regression analysis of predictors for retinal detachment during follow-up after IOFB removal

Variable		Univariate logistic regression		Multivariate logistic regression	
		OR (95% CI)	*P* value	OR (95% CI)	*P* value
**Route of extraction (Pars plana)**		**3.789 (0.686–20.946)**	**0.127**	-	**0.117**
**Impacted IOFB**		**9.722 (1.098–86.103)**	**0.041**	**11.515 (1.096–120.948)**	**0.042**
**PVD (No PVD)**		**0.421 (0.076–2.327)**	**0.321**	**-**	**0.888**
**Entrance wound site**	**Corneoscleral**	**1.360 (0.131–14.165)**	**0.797**	**-**	**0.498**
	**Scleral**	**3.400 (0.489–23.652)**	**0.216**		**0.520**
**Silicon endotamponade**		**6.682 (0.756–59.047)**	**0.088**	**-**	**0.947**
**FB longest diameter / mm**		**1.906 (1.101–3.302)**	**0.021**	**2.119 (1.067–4.207)**	**0.032**
**Intra-operative complications**		**13.125 (2.224–77.447)**	**0.004**	**-**	**0.050**

The median time for silicone oil removal in both groups was 12 weeks (IQR 10–14 weeks), with no statistically significant difference between the groups (*P* = 1).

Patients who developed retinal detachment during the follow-up period were readmitted and pars plana vitrectomy was performed to reattach the retina. By the end of 12-month follow-up period, all eyes had an attached retina.

### Visual outcomes

**Fig. 1:**
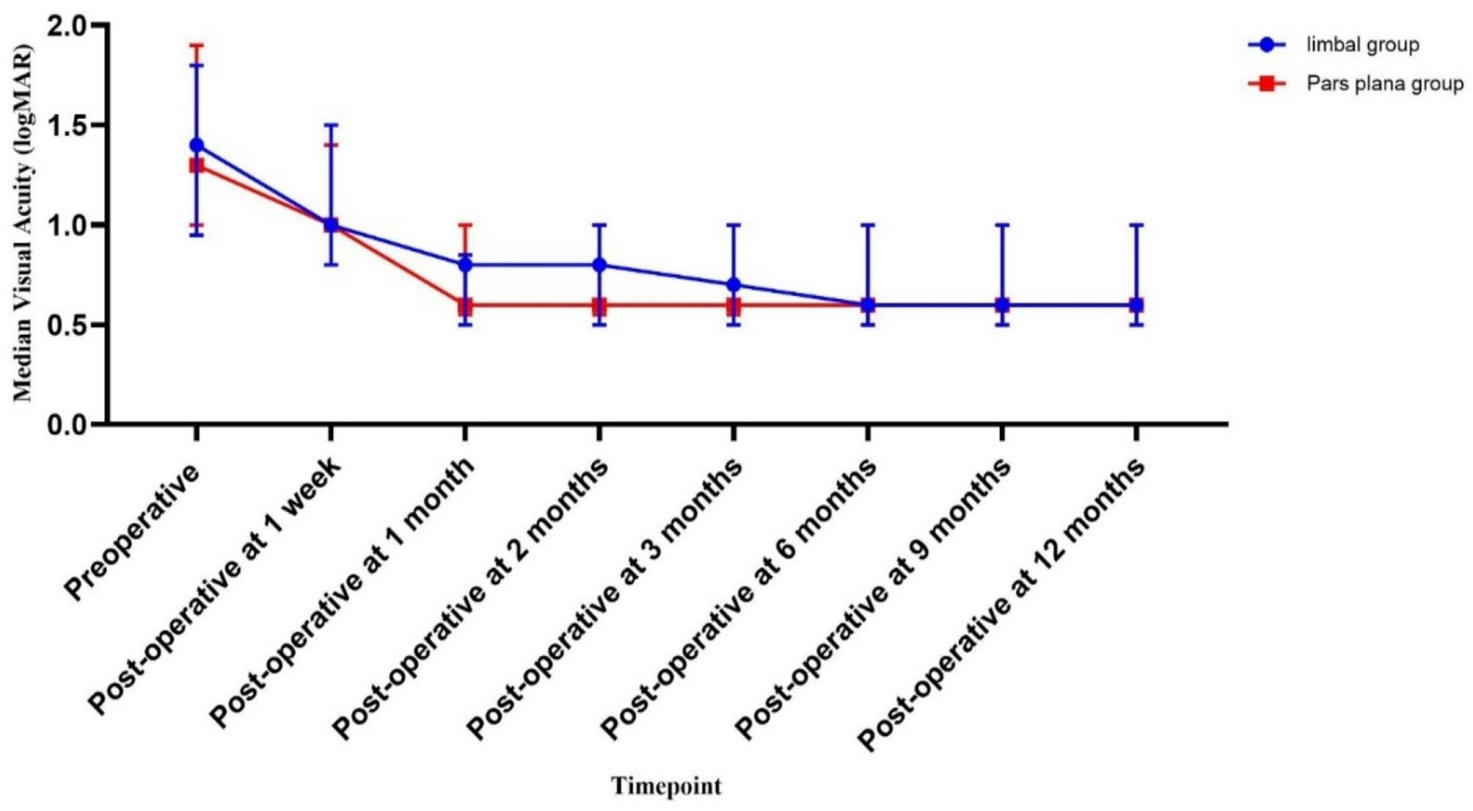
Change in median VA measured by logMAR with IQR range

at baseline, the median logMAR visual acuity was 1.4 (IQR 0.95–1.8) at the limbal group and 1.3 (IQR 1 -1.9) in the pars plana group with no statistically significant difference between both groups (*P* = 0.820). Visual acuity was reassessed at one week postoperatively, then monthly for the first 3 months and then every 3 months thereafter till one year after PPV and IOFB extraction. A statistically significant improvement of the median BCVA from baseline was noticed at the one-month timepoint follow-up in both groups (˂0.001). No significant visual gain was observed after this timepoint (˃ 0.05). Moreover, there was no statistical difference between the 2 groups in BCVA at each follow-up visit (all P-values were ˃ 0.05) (Fig. 1).

Patients with a visual acuity worse than 0.8 logMAR at one year were considered to have a poor visual outcome. To identify associated risk factors, univariate logistic regression analysis was performed on nine potential predictors: route of IOFB extraction (limbal vs. pars plana), preoperative endophthalmitis, entry wound site, median entry wound length, median longest diameter of the foreign body, retinal reattachment surgery (for cases who developed retinal detachment occurring during follow-up), foreign body location (macular vs. extramacular), corneal decompensation, and preoperative visual acuity ≤ 1.6 logMAR. In the univariate analysis, median entry wound length, median foreign body longest diameter, retinal reattachment surgery, macular location of the foreign body, corneal decompensation, and preoperative visual acuity ≤ 1.6 logMAR were all significantly associated with a higher odds ratio for poor visual outcome (*P* < 0.05 for each). Subsequent stepwise multivariate logistic regression analysis identified median entry wound length (*P* = 0.011) and preoperative visual acuity ≤ 1.6 logMAR (*P* = 0.005) as independent risk factors for poor visual outcome at one year following PPV and IOFB extraction (Table 4).

**Table 4:** Univariate and multivariate logistic regression analysis of predictors of poor visual outcome (worse than 0.8 logMAR) one year after IOFB removal

Variable		Univariate logistic regression		Multivariate logistic regression	
		OR (95% CI)	*P* value	OR (95% CI)	*P* value
**Route of extraction (limbal)**		**0.947 (0.277–3.242)**	**0.931**	-	**0.090**
**Endophthalmitis**		**2.067 (0.484–8.829)**	**0.327**	**12.053 (1.235–117.469)**	**0.188**
**Entry wound site**	**Cornea**	**0.786 (0.125–4.923)**	**0.797**	**-**	**0.987**
	**Corneoscleral**	**0.400 (0.026–6.176)**	**0.512**		**0.643**
**Median wound length (IQR)/mm**		**2.697 (1.440–5.051)**	**0.002**	**2.919 (1.283–6.642)**	**0.011**
**FB longest diameter / mm**		**2.251 (1.301–3.893)**	**0.004**	**1.781 (0.937–3.387)**	**0.078**
**Retinal reattachment surgery**		**98.08 (5.025–1914.19)**	**0.003**	**-**	**-**
**FB location in relation to macula**		**6.296 (1.260- 31.465)**	**0.025**	**-**	**0.653**
**Corneal decompensation**		**32.14 (1.599–646.17)**	**0.023**	**-**	**-**
**Preoperative VA ≤ 1.6 logMAR**		**21.75 (4.016–117.78)**	**< 0.001**	**25.251 (2.694–236.70)**	**0.005**

## Discussion

Ocular trauma is a significant cause of vision impairment and disability worldwide, but advancements in microsurgical techniques, and trauma management have greatly improved outcomes.

After primary wound repair, patients were reevaluated and those who met the study’s eligibility criteria were enrolled, randomized and scheduled for a planned 23-gauge pars plana vitrectomy (PPV) and intraocular foreign body (IOFB) extraction following a deferment period of 10–14 days. This two-stage approach was selected to allow for better wound healing, ensuring a watertight globe, facilitating the development of spontaneous posterior vitreous detachment (PVD), and optimizing the surgical set up. Penetrating ocular trauma induces mechanical disruption, which can predispose the vitreous gel to spontaneous detachment from the retina. Additionally, trauma-related intraocular hemorrhage or inflammation may further promote PVD development. Earlier surgical intervention was performed in cases complicated by endophthalmitis or secondary glaucoma during the waiting period, given the risk of rapid disease progression and potential vision loss.

To evaluate the potential impact of the extraction route on the development of postoperative retinal detachment (RD), only cases with an attached retina at presentation were included to minimize confounding factors. Identifying a safer surgical approach could help reduce the likelihood of secondary interventions and their associated complications.

In our study, the overall incidence of postoperative retinal detachment (RD) following pars plana vitrectomy (PPV) and intraocular foreign body (IOFB) extraction was 15.7% (8 eyes), with 2 cases (7.7%) occurring in the limbal group and 6 cases (24%) in the pars plana group. Although the RD rate was higher in the pars plana group, the difference was not statistically significant, likely due to the limited sample size. These findings fall within the range of RD rates reported in the literature, which vary from 6 to 47%. Notably, more recent studies have reported lower rates, likely reflecting advancements in surgical techniques and instrumentation.

Soliman et al. (2021) reported a 6% postoperative RD rate in a cohort of 33 eyes following IOFB removal via the limbus using the iris shelf technique [5]. Similarly, Yuksel et al. (2015) found a 5.5% incidence of RD after extracting IOFBs through the pars plana using 23-gauge vitrectomy in 36 eyes [3]. In contrast, Ehlers et al. (2008) reported a substantially higher RD rate of 47% in a retrospective series of 96 eyes, this elevated rate may be attributed to the use of wide-gauge instrumentation and external magnets, which were commonly employed at that time [13]. This trend toward lower RD rates in more recent reports supports the view that advancements in microincisional vitrectomy, improved visualization systems, and refined surgical approaches have contributed to safer IOFB removal with reduced complications.

In the present study, predictors for the development of postoperative retinal detachment were impacted IOFB, *P* = 0.042 and FB longest diameter, *P* = 0.032, each 1 mm increase in the FB longest diameter was associated with a more than twofold increase in the odds of developing retinal detachment (OR = 2.119, 95% CI: 1.067–4.207), This finding underscores that even small increments in FB size meaningfully elevate the risk of postoperative retinal detachment.

Although all impacted IOFBs were surrounded intraoperatively by two rows of endolaser photocoagulation prior to removal, their initial presence remained an independent predictor of postoperative retinal detachment. This finding suggests that the risk associated with IOFB impaction is not solely due to surgical manipulation during extraction but may also reflect underlying retinal damage from the initial trauma, both potentially triggering a cascade of events that contribute to the development of RD. Predictors for the development of postoperative RD were analyzed in few studies. Parke et al., (2012) at the Bascom Palmer Eye Institute, reported a postoperative retinal detachment rate of 11% in a cohort of 102 eyes without preoperative RD. They identified the presence of retinal impact sites and endophthalmitis as significant risk factors for RD development following IOFB extraction [14], El-Asrar et al., (1998) identified intraocular foreign body size of more than 4 mm, and the presence of endophthalmitis as predictors for the development of postoperative retinal detachment [15]. While Markan and associates (2024) reported that zone of injury, route of extraction, impacted IOFB, size of IOFB, tamponade and endophthalmitis were none predictive of the anatomical results. In the present study, the route of extraction (pars plana versus limbal) did not emerge as significant predictor, neither in the univariate nor in the multivariate analysis [16].

In the present study, the median preoperative logMAR visual acuity was 1.3 (equivalent to 0.05 decimal or 3/60 Snellen). A statistically significant improvement in median best-corrected visual acuity (BCVA) from baseline was observed at the one-month follow-up in both groups with no significant visual gain noted beyond this timepoint. Additionally, there was no statistically significant difference in BCVA between the two groups at any follow-up visit. At the one-year follow-up, 72.5% of patients achieved a visual acuity of ≥ 0.8 logMAR (0.16 decimal, 6/36 Snellen), while 27.5% achieved a VA of < 0.8 logMAR (≤ 0.1 decimal, 6/60 Snellen).

The results are generally comparable to those reported in previous studies, Al-Ani et al. (2025) found that 75% of 32 eyes with IOFBs achieved a final visual acuity of ≥ 1.0 logMAR (0.1 decimal, 6/60 Snellen), with 25% improving to < 1.0 logMAR (0.05 decimal, 3/60 Snellen) [17]. Hapca et al. (2022), in a study of 56 eyes, reported that 46.4% of patients achieved a final visual acuity of ≥ 1 logMAR (0.1 decimal, 6/60 Snellen), while 53.6% achieved a VA of < 1 logMAR (≤ 0.05 decimal, 5/60 Snellen) [18]. Ratanapakorn et al. (2021), in a large series of 359 eyes, reported that 36.2% of patients achieved a visual acuity of ≥ 0.8 logMAR (0.16 decimal, 6/36 Snellen), while 63.8% achieved a VA of < 0.8 logMAR (≤ 0.1 decimal, 6/60 Snellen). Their cohort included a notable proportion of patients (15%) with no light perception preoperatively, possibly influencing the overall outcome [19]. Nicoară et al. (2015) reported that in 21 cases with IOFBs 8 patients, 47.6% achieved VA ≥ 1 logMAR (0.16 decimal, 6/60 Snellen) and 52.4% of patients achieved VA < 1 logMAR (≤ 0.05 decimal, 5/60 Snellen) [20]. Compared to prior studies, our visual outcomes appear favorable, possibly reflecting effective surgical techniques and careful case selection. However, differences in baseline characteristics and IOFB-related complications across studies must be considered when interpreting these comparisons.

In the present work, stepwise multivariate logistic regression identified two independent significant predictors of poor visual outcome: median wound length (*P* = 0.011) and preoperative visual acuity ≤ 1.6 (*P* = 0.005). Regarding median wound length, the odds of a poor visual outcome increased by nearly 3 times for each 1 mm increase in wound length, suggesting that longer wounds may inflict more structural tissue damage which negatively impact visual recovery. Preoperative poor vision (defined as VA ≤ 1.6 logMAR equivalent to 5/200) was a strong predictor of poor postoperative visual outcome. Patients with poor baseline vision had over 25 times higher odds of remaining with poor vision postoperatively. This agrees with previous studies which have consistently identified baseline visual acuity as a strong prognostic indicator in ocular trauma cases involving IOFB [18, 19, 21]. The route of intraocular foreign body (IOFB) extraction, limbal versus pars plana, was not identified as a significant predictor of poor visual outcome in either univariate or multivariate logistic regression analysis.

In the present study, endophthalmitis was not found to be a significant risk factor for poor visual outcome. This finding may be explained by the study design, which excluded patients who initially presented with traumatic endophthalmitis associated with IOFBs. Additionally, all patients were closely monitored during the deferment period, and those who developed signs suggestive of endophthalmitis underwent timely pars plana vitrectomy and IOFB removal. These measures likely minimize the impact of infection on visual prognosis. The literature on this topic remains inconsistent; while some studies have identified endophthalmitis as a significant predictor of poor postoperative visual outcomes [18, 19], others have not demonstrated a clear association [20]. Our results align more closely with the latter, suggesting that with appropriate case selection and prompt intervention, the detrimental effect of endophthalmitis on visual outcomes may be reduced.

The route of IOFB extraction did not influence the structural or functional outcomes, suggesting that both limbal and pars plana approaches are viable when appropriately selected. Surgical planning should therefore prioritize IOFB characteristics and associated ocular trauma rather than the extraction route alone. Our findings indicate that larger, impacted IOFBs significantly increase the risk of postoperative retinal detachment, while poor presenting visual acuity and longer wound length are strong predictors of suboptimal visual recovery. Notably, our study was conducted prospectively with predefined inclusion and exclusion criteria, providing a structured approach that complements and adds to the predominantly retrospective literature available and discussed. These methodological strengths enhance the reliability of our findings and support a more individualized, risk-adapted approach to IOFB management. Future studies with larger cohorts and longer follow-up may further clarify these relationships and help refine surgical strategies in complex ocular trauma.

## Data Availability

The datasets generated and/or analyzed during the current study are available from the corresponding author upon reasonable request.
